# Postoperative Euglycemic Ketoacidosis in Type 2 Diabetes Associated with Sodium-Glucose Cotransporter 2 Inhibitor: Insights Into Pathogenesis and Management Strategy

**DOI:** 10.7759/cureus.15533

**Published:** 2021-06-08

**Authors:** Harshith P Chandrakumar, Seeta Chillumuntala, Gurcharan Singh, Samy I McFarlane

**Affiliations:** 1 Department of Medicine, State University of New York (SUNY) Downstate, Health Science University, Brooklyn, USA

**Keywords:** sodium-glucose cotransporter 2 (sglt-2) inhibitors, ertugliflozin, euglycemic diabetic ketoacidosis, postoperative period, adverse drug reaction, diabetes type 2

## Abstract

Euglycemic diabetic ketoacidosis (eu-DKA) is an uncommon and serious adverse event associated with the use of sodium-glucose cotransporter (SGLT-2) inhibitors. It is a state of increased anion gap metabolic acidosis with ketosis but in the setting of normal serum glucose levels. Diagnosis of this serious entity could easily be missed given the non-specific symptoms and the normal glucose measurements.

This ketogenic state can be triggered by various stressors including infection, surgery, myocardial infarctions, omission of insulin dosage, as well as low carbohydrate diet. In this report, we present a case of eu-DKA in a 68-year-old woman with type 2 diabetes that occurred in the postoperative period of glaucoma surgery. She was started shortly before surgery on SGLT-2 inhibitor (ertugliflozin). While the diagnosis was initially missed, it was subsequently confirmed when she presented with reduced appetite, generalized fatigue, and constipation. Ertugliflozin was discontinued, and she was successfully treated with conservative management and without insulin drip.

This case highlights the need to consider the diagnosis of eu-DKA in patients treated with SGLT-2 inhibitors since the diagnosis could easily be missed especially in the postoperative period with the non-characteristic symptomatology and normoglycemia.

## Introduction

Sodium-glucose cotransporter (SGLT-2) inhibitors that are a relatively newer class of antidiabetic medications have emerged as important therapeutic agents that reduce cardiovascular events, hospitalization from heart failure, as well as the progression of chronic kidney disease (CKD) in both diabetic and non-diabetic populations. The therapeutic indications for these agents are rapidly changing the guidelines of the American Diabetes Association (ADA), American College of Cardiology (ACC), as well as other major national and international organizations [1-6]. This class of anti-diabetic drugs specifically acts on the proximal renal tubules, causing a reduction in serum blood glucose through glycosuria [2]. One of the serious adverse effects of SGLT-2 inhibitors is the occurrence of euglycemic diabetic ketoacidosis (eu-DKA) with an incidence of ≤ 0.3% from recent trials [6-8]. To date, there are four Food and Drug Administration (FDA)-approved SGLT-2 inhibitor drugs that have been approved for type 2 diabetes mellitus, which include canagliflozin, dapagliflozin, empagliflozin, and ertugliflozin. Ertugliflozin is a much newer agent with around 20 cases of eu-DKA reported in the literature, including the Vertis CV trial [8-11]. Of note also, these agents have been increasingly used off-label in type 1 diabetes with multiple reports on eu-DKA in this susceptible population [9].

In this report, we present a case of a 68-year-old woman with type 2 diabetes who developed eu-DKA after initiation of ertugliflozin therapy and was precipitated by recent eye surgery. Of importance, the diagnosis was missed during the initial visit given the non-specific nature of the symptoms and the normal glucose values obtained during the post-operative period. This patient was treated conservatively without insulin drip, indicating that she has insulin secretory capacity and corollary to the management of alcoholic ketoacidosis, which is another sub-type of euglycemic ketoacidosis. We also provide a literature review on the diagnosis and preventive measures of this potentially serious adverse effect of SGLT-2 inhibitors.

## Case presentation

Initial admission

The patient is a 68-year-old woman with hypertension, type 2 diabetes, and recent right eye surgery for glaucoma two days prior to admission when she presented with a right-sided headache with blurry vision and slowed speech lasting for a few hours. The symptoms spontaneously resolved during the initial emergency room interview. There was no weakness, incontinence, syncopal episodes, or trauma. Physical exam showed a fixed dilated pupil with decreased visual acuity likely secondary to surgery with no focal neurological deficits. She was admitted for workup of transient ischemic attack.

Of note, she was initiated on ertugliflozin right before the surgery. Initial labs (Table 1) showed an anion gap (AG) of 27 mmol/L and bicarbonate (HCO3-) of 8 mmol/L with a blood glucose of 147 mg/dl. A computed tomography (CT) scan of the head, as well as CT angiography of the head and neck, showed no abnormalities. Clinically improved with symptom resolution, she was discharged the next day to continue with home medications. Her medications at the time included simvastatin, metoprolol, and ertugliflozin 15 mg once daily.

**Table 1:** Laboratory values during admission and discharge

Labs	Initial admission	Repeat admission	On discharge
Hemoglobin (g/dL)	14.4	14.1	12.3
White blood cells (K/uL)	8.16	10.02	6.61
Platelets (K/uL)	271	197	203
Sodium (mmol/L)	131	132	137
Potassium (mmol/L)	4.9	4.3	3.9
Chloride (mmol/L)	100	94	101
CO2 (mmol/L)	8	15	21
Blood urea nitrogen(mg/dL)	19	13	12
Creatinine(mg/dL)	1.2	0.9	0.7
Anion gap(mmol/L)	27	27	19
Blood glucose (mg/dL)	147	110	151

Repeat admission

The patient presented eight days later with decreased appetite, generalized malaise, and constipation. The physical exam was unremarkable. However, labs (Table 1) showed a persistently elevated AG of 23, HCO3- of 15, beta-hydroxybutyrate(BHB) of 4.89, and pH of 7.36 on blood gas measurement, together with a lactate of 1.2 mmol/L, hemoglobin A1c of 12.8%, and blood glucose of 110 mg/dl. Urinalysis showed glucose >500 mg/dL and ketones of 80 mg/dL (Table 2). She had normal kidney function and denied any substance or alcohol use. Electrocardiogram (EKG) at the time showed new T wave inversions in lead V3, but repeat troponins remained negative. CT of the abdomen with intravenous contrast showed a large stool burden in the rectum and sigmoid colon, and a fleet enema was administered. Upon further questioning, the patient reported non-compliance with anti-diabetic agents, including metformin, with ertugliflozin being the first agent she took consistently.

**Table 2:** Additional laboratory values

Labs	Result
Lactate (mmol/L)	1.2
Hemoglobin A1c (%)	12.6
Beta-hydroxy butyrate (mmol/L)	4.89
Urine pH	5
Urine glucose (mg/dL)	>500
Urine ketones (mg/dL)	80
Urine specific gravity	1.021
Urine nitrite	negative
Urine leucocyte esterase	negative
Venous blood gas: pH	7.36
pCO2 (mmHg)	37.7
pO2 (mmHg)	30.3
HCO3 (mmHg)	21

Management

The diagnosis of mild eu-DKA secondary to the SGLT-2 inhibitor was made. An endocrinology consult was sought and was advised to stop ertugliflozin and continue supportive management with intravenous fluids and insulin aspart coverage. The patient was also administered 2 liters of Ringer’s lactate solution and was continued on 100 mL/hour continuous infusion. Diet was re-initiated as tolerated. Soon after ertugliflozin was held, the patient's appetite and bowel movements improved. She was observed for symptomatic improvement and on Day 2 of hospitalization, AG improved to 19 and HCO3- to 21. Blood glucose during the hospital course remained <140 mg/dL and did not require any additional insulin. On the last day of hospitalization, the patient noted having the ‘best meal’ in two weeks as opposed to her reduced appetite previously. Upon discharge, ertugliflozin was held; she was initiated on sitagliptin and discharged home with endocrinology follow-up.

## Discussion

SGLT-2 receptors are located in the early S1 segment of the proximal renal tubules as opposed to the SGLT-1 receptors that are located in the S2, S3 segment. Under normal physiology, 80%-90% of the glucose reaching the proximal tubules is absorbed by the SGLT-2 receptors, and 10-20% is absorbed by the SGLT-1 receptors [2]. SGLT-2 receptors are also located in the alpha 2 pancreatic cells [3]. Being a co-transporter, they absorb glucose with every molecule of sodium into the blood passively.

In nondiabetic individuals, the renal threshold for glucosuria is 180 mg/dL, with most of the glucose being reabsorbed in the proximal renal tubules [2]. But in the diabetic population, the renal threshold is increased, partly due to upregulation of SGLT-2 receptors contributing to increased glucose co-transport leading to hyperglycemia [4]. SGLT-2 inhibitor blocks this mechanism causing reduction of hyperglycemia, independent of insulin and thereby avoiding hypoglycemic episodes. This is manifested by increasing glucosuria and improving insulin sensitivity in peripheral tissues [2]. Although these drugs improve the glycemic index, the occurrence of eu-DKA should be considered.

Diabetic ketoacidosis (DKA) includes hyperglycemia (serum glucose >250 mg/dL), acidosis (arterial pH <7.3 and bicarbonate <15 mEq/L) and ketosis (ketonuria or ketonemia) [5]. Whereas euglycemic DKA is a triad comprising of increased anion gap acidosis, evidence of ketosis while being normoglycemic (serum glucose < 250 mg/dL).

The incidence of DKA associated with SGLT-2 inhibitors has been ≤0.3% in the trials involving empagliflozin, dapagliflozin, and ertugliflozin [6-8]. A meta-analysis by Burke et al. looked at 34 cases of DKA caused by SGLT-2 inhibitors of which 25 were of type 2 DM patients [9]. Ertugliflozin was FDA approved in 2017 and is the newest of the SGLT-2 inhibitors. There have been only two reported cases of ertugliflozin-induced euglycemic DKA, to our knowledge, thus far per literature review [10-11]. In the Vertis CV trial that looked at the cardiovascular effects of ertugliflozin, 5,490 cases were included. Of which, 19 cases had DKA, seven occurring in the lower dose group (5 mg) and 12 in the higher dose group (15 mg) [8]. Our patient was prescribed a higher dose of 15 mg, which is consistent with the notion that higher doses are more prone to the development of eu-DKA.

Most patients developing euglycemic DKA may have a precipitating event. The commonly reported factors include surgery, infections including COVID-19, pericarditis, concurrent ketogenic/low carbohydrate diets, a recent change of diabetic regimen with reduction or omission of insulin dosages, nausea and vomiting, gastroparesis, pregnancy, and acute pancreatitis [12-19]. Diagnosis is often delayed or missed in these settings, like in our case presentation. The reported blood glucose in these cases ranged from 127-212 mg/dL [12-19].

The pathogenesis of eu-DKA is not entirely certain. But, it has been hypothesized that SGLT-2 inhibitors lead to increased glycosuria and reduced serum blood glucose levels, with a shift in metabolism towards the utilization of gluconeogenesis and lipolysis. As there is persistently normal glucose, insulin secretion is reduced to a minimum [2-3]. This phenomenon has the following impact: 1) Increased lipolysis within adipose tissues and production of free fatty acids [20]; 2) Ketone body production in the liver from the transport of fatty acids into the hepatic mitochondria [20]; and 3) SGLT-2 inhibition also stimulates glucagon production, which further induces hepatic production of ketone bodies and resultant ketogenesis [3].

Patients on SGLT-2 inhibitors have reduced glycogen stores and any inciting event can precipitate eu-DKA. Physiologic stressors like surgery and infection can increase stress hormones like cortisol, adrenaline causing further insulin resistance and protein catabolism from alpha and beta receptor stimulation [15,20]. Furthermore, SGLT-2 inhibitors can decrease the appetite, as in our patient, and can potentiate the ketogenic state (starvation ketosis).

Typically, from most of the reported cases, eu-DKA has been managed similar to hyperglycemic DKA with intravenous insulin, fluids, and frequent monitoring of serum electrolytes until anion gap acidosis resolution [12-19]. But, patients could be conservatively managed like in our case. Our patient was initiated on lactated Ringer’s fluids and a diabetic diet. The AG spontaneously resolved with close monitoring and the patient felt significantly better. This management is particularly possible in type 2 diabetes patients with somewhat reserved endogenous insulin secretory capacity, as with the case management of alcoholic ketoacidosis, a sub-type of euglycemic ketoacidosis. This management strategy, however, would be contraindicated in type 1 diabetes where insulin deficiency is the hallmark of the disease.

Important learning points

1) The diagnosis of eu-DKA in patients on SGLT-2 inhibitors can be easily missed or delayed, given the non-specific symptomatology together with normal blood glucose. Therefore, keeping this diagnostic entity in mind is quite critical for early diagnosis and management of this potentially serious adverse event.

2) Higher dose of ertugliflozin is probably more prone to eu-DKA development.

3) Insulin drip is not required in all cases, particularly in type 2 diabetes with partially preserved insulin secretory capacity. Patients can initially be conservatively managed with fluids and initiation of dietary management.

4) With rapidly increasing indications for the use of SGLT-2 inhibitors and increasing usage in heart failure populations, potential acidosis should be considered as it poses a particularly high risk of increased mortality in acute heart failure patients.

## Conclusions

There has been a growing indication for SGLT-2 inhibitors given the evidence from major clinical trials of reduction of cardiovascular events, hospitalization for heart failure, as well as the progression of CKD in both diabetic and nondiabetic populations. Although the incidence of eu-DKA is relatively low, it should be considered especially in patients with risk factors that could precipitate eu-DKA, including those with infection, myocardial infarction, trauma, as well as postoperatively as in our case presentation.
